# A three-dimensional vision measurement method based on double-line combined structured light

**DOI:** 10.1038/s41598-023-46176-y

**Published:** 2023-10-31

**Authors:** Mingze Wang, Qiucheng Sun, Changbo Gao, Zeming Ren, Weiyu Dai

**Affiliations:** College of Computer Science and Technology, Changchun Normal University, Changchun, 130032 China

**Keywords:** Engineering, Optics and photonics

## Abstract

In this paper, a structured light vision measurement method using a scanning laser line and a positioning laser line is proposed. The novel method enables the scanning laser plane to slide along a slide rail while maintaining intersection with the positioning laser plane, eliminating the need to determine the scanning direction and moving step. During the measurement process, the laser plane equations need to be recalibrated for each new position, so a real-time calibration method is given. Initially, the geometric barycenter method is employed to detect the subpixel coordinates of the light stripe intersection point. Subsequently, these coordinates are projected into the camera coordinate system using the initial equations of the positioning laser plane. Finally, leveraging the normal information of the initial equation of the scanning laser plane and the three-dimensional coordinates of the light stripe intersection point, the real-time calibration of the scanning laser plane equations can be accomplished. The proposed method enables the three-dimensional reconstruction of objects, and its accuracy is verified through measurements on gauge blocks. Experimental results demonstrate that this method achieves precise and stable three-dimensional reconstruction of object surface shape.

## Introduction

With the swift progress in modern manufacturing and technology, three-dimensional (3D) reconstruction techniques have garnered significant attention in both research and practical applications, encompassing fields such as machine vision, biomedical science, entertainment, and contemporary industry^1,2^. Especially within the manufacturing sector, there has been a rising demand for measurements with high precision and efficiency. This has catalyzed the evolution and refinement of numerous non-contact 3D measurement techniques, including structured light measurement, 3D laser scanning, and Time-of-Flight (TOF) camera technologies^3^.

Structured light measurement technology captures precise 3D information of an object by projecting specific light patterns onto its surface and subsequently analyzing the reflected patterns or distortions. Because of its suitability for complex-shaped objects, combined with its high precision, speed, cost-effectiveness, and stability, structured light measurement emerges as a preferred method for close-range, small-scale measurement scenarios in the non-contact measurement domain^4^. In contrast, 3D laser scanning technology utilizes a laser beam to scan a target object, processing its reflected light to reconstruct the object’s surface topology. Although this technology is suitable for large-scale and long-distance measurement scenarios, for smaller research groups, high-precision instruments may be economically unaffordable. More cost-effective equipment might compromise on accuracy, and may not be as effective as structured light measurement methods in low-cost, small-scale measurement applications^5–7^. TOF (Time-of-Flight) cameras operate based on the time it takes for light to propagate on the surface of an object and measure distance, offering advantages in speed and measurement range. However, in close-distance, small-scale measurement scenarios, its stability and measurement accuracy may not be as good as structured light measurement methods^8–10^.

Structured light measurement techniques can be broadly divided into three categories: point-structured, surface-structured, and line-structured light. The point-structured light measurement technique projects a laser onto an object’s surface, producing a laser dot. By analyzing the positional shift of this laser dot on a photosensitive element, the depth information of the dot can be deduced^11^. However, this method is limited to measuring a finite number of positions at any given moment. To comprehensively capture the structural intricacies of the entire surface, scanning must be done point by point or line by line. Consequently, the data acquired for an object’s surface details is often insufficient.

On the other hand, the surface-structured light measurement technique employs a projector to cast raster images onto the object’s surface. By analyzing the modulated raster image influenced by the object’s surface, the 3D contour of the surface can be directly determined^12–16^. This approach obviates the need for scanning, a stark contrast to the point-structured light method. However, the act of raster projection requires both the encoding of the projection pattern and the decoding of the captured image, thereby amplifying the intricacy of image processing^17–20^.

The line-structured light measurement technique operates on the principle of laser triangulation. Initially, a linear laser is cast onto the surface of the object in question. Following this, an extraction algorithm identifies and pinpoints the two-dimensional (2D) coordinates of the light stripe’s central point within the image. By mapping these 2D coordinates into the camera’s coordinate system, and considering the pre-calibrated vision system parameters, the spatial coordinates of this central point can be calculated. This approach captures a more extensive set of data in a single pass compared to the point-structured light technique, ensuring heightened efficiency^21^. When set against the surface-structured light technique, the line-structured method boasts more straightforward image processing, given that each image contains just a singular light stripe pattern.

To capture the full 3D profile of the object under measurement, the laser plane must integrate with at least one mobile unit equipped with scanning capabilities. This integration facilitates the laser plane’s movement across the object, following a predefined scanning trajectory. Utilizing the mobile device, combined with the outcomes of the system calibration, data from various images can be consolidated within a singular coordinate system. This process enables the acquisition of point cloud data representing the surface of the measured object^5,22,23^.

Coordinate measuring machines (CMM)^24–26^ are commonly employed as mobile devices. When the 3D scanning system is mounted on the CMM, the data points are directly transformed into 3D data within the CMM coordinate system. Subsequently, a curved surface is constructed by gridding the point cloud. Since the final measurement is performed in the CMM frame, the target’s position in the CMM frame is measured using the trigger probe to derive the conversion from the world coordinate system to the global coordinate system of the CMM. Usually, using contact probes to identify the target orientation in the CMM global frame makes the calibration process inconvenient and not facilitating. Therefore, some methods are proposed without mobile control devices, such as handheld laser scanning^27,28^. However, although these methods mitigate the precision demands of the scanning device, they tend to accumulate measurement errors continuously, and the methods’ performance limits the measurement accuracy.

To simplify the calibration and scanning operations, in the experiments given in the references^29–33^, a linear projector was placed perpendicular to the object surface (x-axis), and the camera was aligned at an oblique angle. In this configuration, the object was fixed on a platform that moved the object along the x-axis (slide rail). However, in practice, the perpendicular relationship between the line laser projector and the object surface is difficult to guarantee and can affect the final measurement accuracy. Therefore, in reference^34^, the above specific vertical relationship was not required, and a relevant algorithm to determine the scanning direction was given to further improve the 3D reconstruction accuracy.

To enhance the measurement accuracy of the system, various studies have incorporated mechanical, specifically stepping motors, as their primary driving mechanisms for scanning motion. For instance, Huang^35^ utilized three stepping motors to drive a console, enabling movement in three dimensions with an accuracy of 0.55 mm. Zhou^36^ employed a stepping motor to drive a table carrying the measured object, facilitating perpendicular movement to the laser plane. Similarly, Li^37^ conducted measurements on a ceramic ball with a radius of 28.494 mm, achieving a measured radius of 28.499 mm with a radius error of only 0.005 mm. By utilizing stepping motors as driving methods, these studies ensured high measurement accuracy. However, it is worth noting that the scanning process of the laser plane controlled by a stepping motor follows a “step → pause → step” pattern, introducing a certain hysteresis. From a strict perspective, such a process deviates from the notion of continuous “scanning,” potentially compromising the real-time efficiency of 3D measurements.

Increasing the number of laser lines can significantly enhance the measurement accuracy and efficiency of line-structured light techniques. For instance, Wu^38^ designed a 3D measurement system utilizing a three-line laser to measure the diameter of a sphere with a measurement error of 0.01 mm. In reference^39^, a multi-line structured light measurement method was employed, achieving a detection accuracy of 99.74% for various computer keyboards. Li^40^ designed a multi-line structured light measurement system, with the measurement error of objects being within 0.4 mm. Reference^41^ presented a multi-line structured light vision technique, realizing a balance between precision and speed in measuring the flatness of steel plates. Gao^42^ employed two three-line lasers, together casting a total of six parallel rays onto the target object. However, it’s pertinent to note that a linear movement of the object remained a requisite to enable efficient scanning measurements.

The multi-line structured light measurement methodology boasts a slew of benefits, encompassing heightened accuracy, amplified efficiency, and a potential for cost savings. Nonetheless, this approach isn’t without its drawbacks. To begin with, prevailing multi-line structured light techniques often handle each light stripe in isolation, devoid of any meaningful interrelation among them. Additionally, despite the multi-line approach, there’s a possibility that a solitary image might fall short in capturing comprehensive data, thereby prompting the need for data collection across multiple images via scanning^43^. Subsequently, the surface point cloud representing the examined object is derived by meticulously aligning and amalgamating the collated scan data.

This paper presents a novel solution to address the aforementioned issues through a double-line combined structured light 3D vision measurement method. The proposed method utilizes two single-line lasers: one acts as a positioning laser plane, while the other serves as a scanning laser plane, with the motion controlled by a slide rail. Throughout the scanning process, the two laser planes consistently intersect each other, eliminating the requirement for a specific spatial relationship, such as perpendicularity. By leveraging the intersection information of the two light stripes and the initial calibration data of the system, real-time calibration of the scanning laser plane is achieved during the motion. This calibration enables the comprehensive measurement of the surface of the object being measured. Importantly, the method proposed in this paper circumvents the need for determining the scanning direction and avoids any efficiency limitations caused by the hysteresis of stepper motors commonly used in measurement systems.

## System structure and measurement steps

### System structure

**Figure 1:**
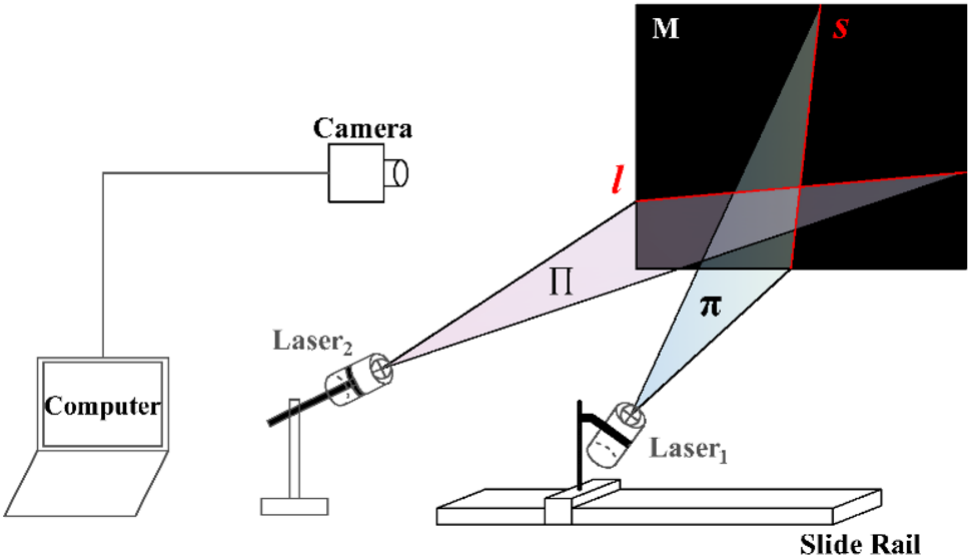
System structure.

The structure of the measurement system is shown in Fig. 1, which consists of two single-line lasers, a slide rail, a camera, and a computer. Laser_1_ (scanning laser) is securely mounted on the slide rail slider. Laser_2_ (positioning laser) remains fixed and maintains a constant intersection angle with Laser_1_. Theoretically, the lasers can intersect at any desired angle. The computer takes charge of controlling the camera to capture continuous images throughout the scanning process, while also performing necessary image processing tasks.

### Measurement steps

**Figure 2:**
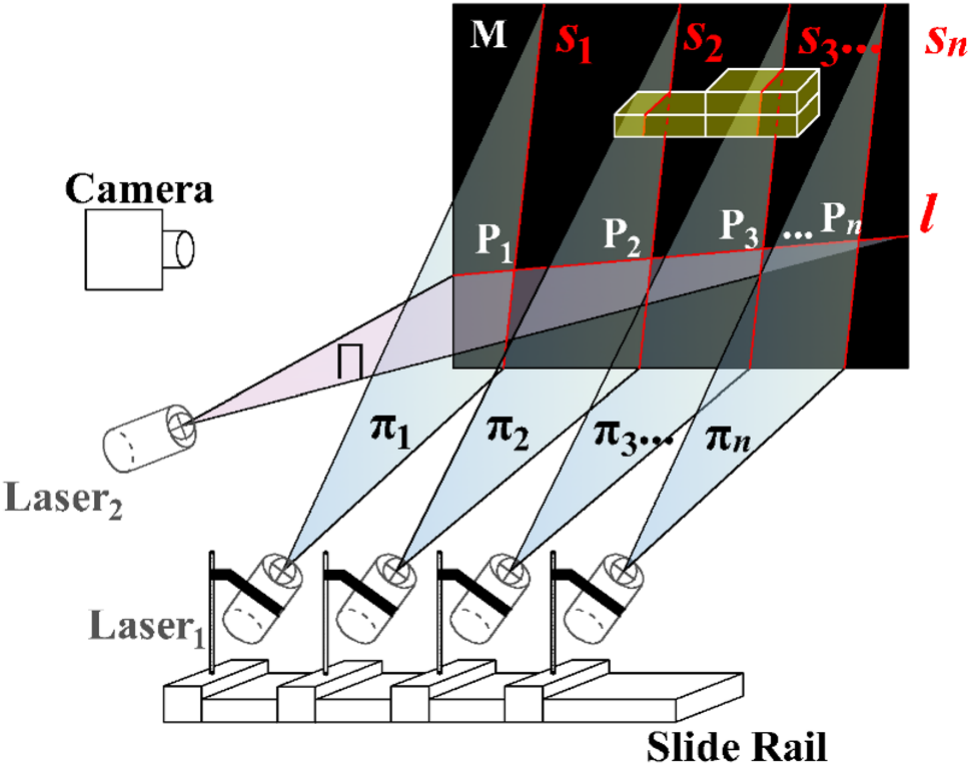
Scanning process of measurement system.

As shown in Fig. 2, the initial laser planes of the system are defined by the scanning laser plane, π_1_, and the positioning laser plane, ∏. The light strip, denoted as *l*, when projected onto the background plane, M, intersects s_1_ at the point labeled P_1_. As the scanning unfolds, a series of subsequent intersection points emerge, represented as P_2_ through P_*n*_.

**Figure 3:**
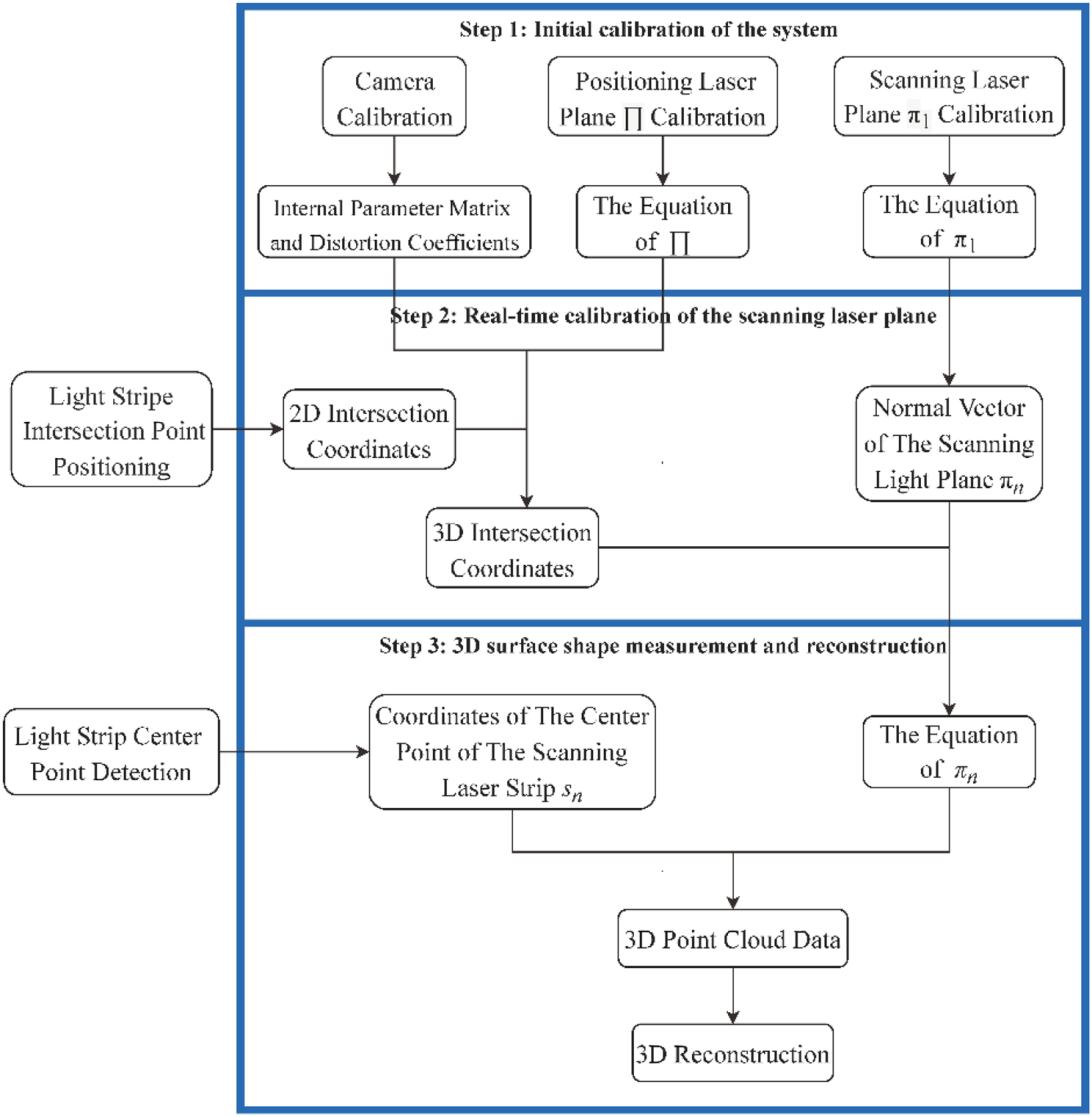
System measurement flow chart.

As shown in Fig. 3, the measurement process can be summarized as the following three steps:

*Step 1: Initial calibration of the system* In this step, the system undergoes calibration procedures, including camera calibration, positioning laser plane calibration, and initial scanning laser plane calibration. This involves obtaining the camera’s internal parameter matrix, distortion coefficients, and equations for both laser planes.

*Step 2: Real-time calibration of the scanning laser plane* During this step, the slider is utilized to facilitate the movement of Laser_1_ along the slide rail in a linear fashion while the camera continuously captures images. The image coordinates of the intersection point P_*n*_ in each image are detected, and their corresponding 3D coordinates are solved by using the calibration information of the camera and the positioning laser plane. Based on the calibration information of the initial scanning laser plane and the 3D coordinates of the intersection point P_*n*_, the equation of the scanning laser plane is calculated at each moment of image capture.

*Step 3: 3D surface shape measurement and reconstruction* In this final step, the center point information of the scanning light stripe image is detected by a light stripe center detection algorithm, and those 3D coordinates are calculated. Subsequently, the 3D surface shape data of the measured object can be obtained by splicing the 3D coordinates of all images, and the 3D reconstruction can be achieved ultimately.

## Calibration and calculation methods

The structure of the double-line combined structured light 3D vision measurement system integrates both a camera and two single-line lasers. Consequently, the initial calibration process encompasses camera calibration to determine the intrinsic parameters, such as the internal parameter matrix and distortion coefficients. Additionally, calibration of the laser plane is carried out to establish the mathematical equations representing the laser planes employed in the measurement system. By precisely determining the equations for the positioning laser plane and the scanning laser plane, the system can accurately identify the intersection points between the laser stripes and the object surface. These initial calibration steps ensure the system’s capacity for accurate and reliable measurements.

### Camera calibration

**Figure 4:**
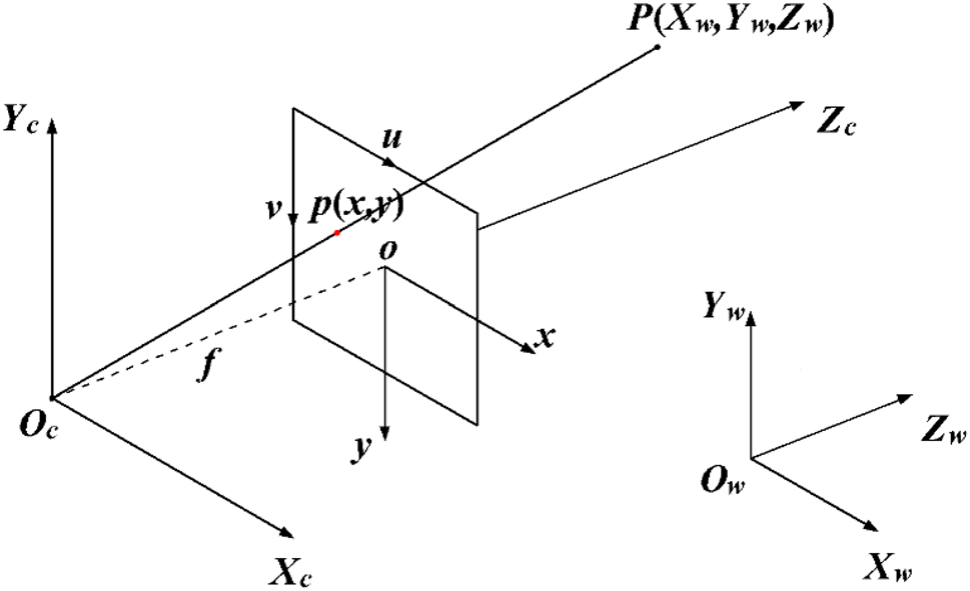
Coordinate system of camera imaging.

The basic principle of camera calibration is that a non-linear relationship exists between the three-dimensional (3D) surface of an object and its two-dimensional (2D) image captured by the camera. The pinhole imaging model is commonly employed to describe the linear relationship of perspective projection while considering lens distortion effects. During camera calibration, four coordinate systems are established to precisely describe the transformation relationships between coordinates. These coordinate systems include the world coordinate system $({X}_{w},{Y}_{w},{Z}_{w})$, the camera coordinate system $({X}_{c},{Y}_{c},{Z}_{c})$, the image physical coordinate system $(x,y)$, and the image pixel coordinate system $(u,v)$, as shown in Fig. 4.

Zhang’s calibration method^44^ is widely recognized as one of the most commonly used camera calibration techniques. This method establishes the world coordinate system by selecting a reference point on the calibration plate, typically the upper-left corner, as the origin. The ${X}_{w}{O}_{w}{Y}_{w}$ plane of the world coordinate system coincides with the plane of the calibration plate, ensuring that the points on the calibration plate have a constant Z-coordinate value of zero. To calculate the camera’s internal parameter matrix $A$, external parameter matrix $R$, translation vector $T$, and distortion coefficient $K$, $N (N \ge 3)$ calibration plate images are captured from different positions, and the corner point coordinates of each image are extracted. In this paper, tangential distortion is added based on Zhang’s calibration model, which makes the calibration model become a non-linear model more in line with high-precision measurement. By utilizing a non-linear optimization function, typically solved using the Levenberg–Marquardt (L–M) algorithm, the camera calibration process can be completed. The internal parameter matrix $A$ and distortion coefficient $K{({k}_{1},{k}_{2},{p}_{1},{p}_{2})}^{T}$ of the camera can be accurately determined.

### Laser plane calibration

In a previous study^45^, a laser plane calibration algorithm utilizing a planar target with a square pattern is proposed. The calibration process involves capturing the target image from a minimum of two distinct spatial positions. By projecting the pixel coordinates of the light stripe’s center point onto the camera coordinate system, a set of 3D coordinates can be obtained. These 3D coordinates are then used to determine the laser plane equations through a fitting procedure.

**Figure 5:**
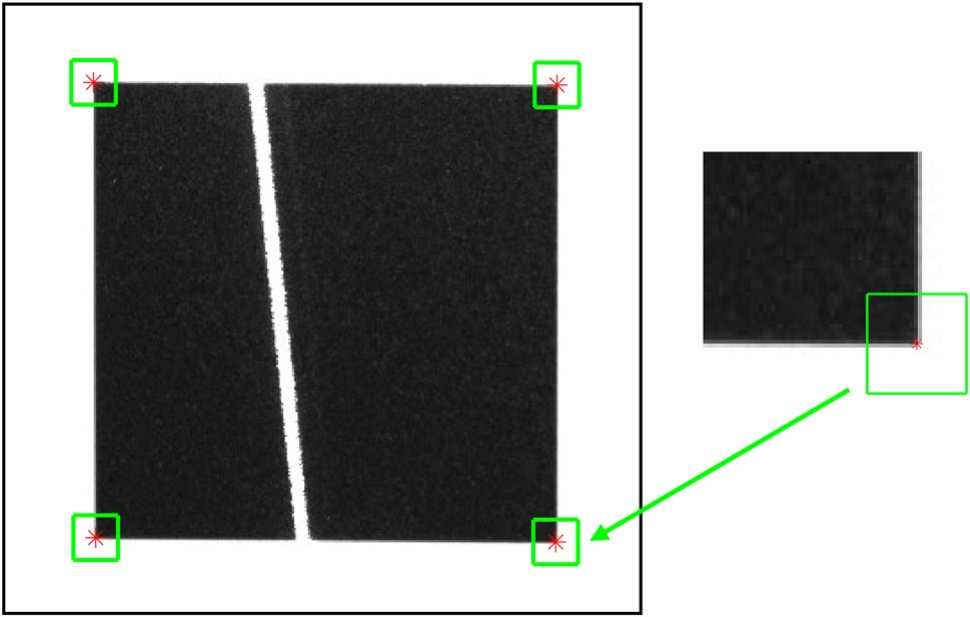
Extraction of corner point coordinates.

**Figure 6:**
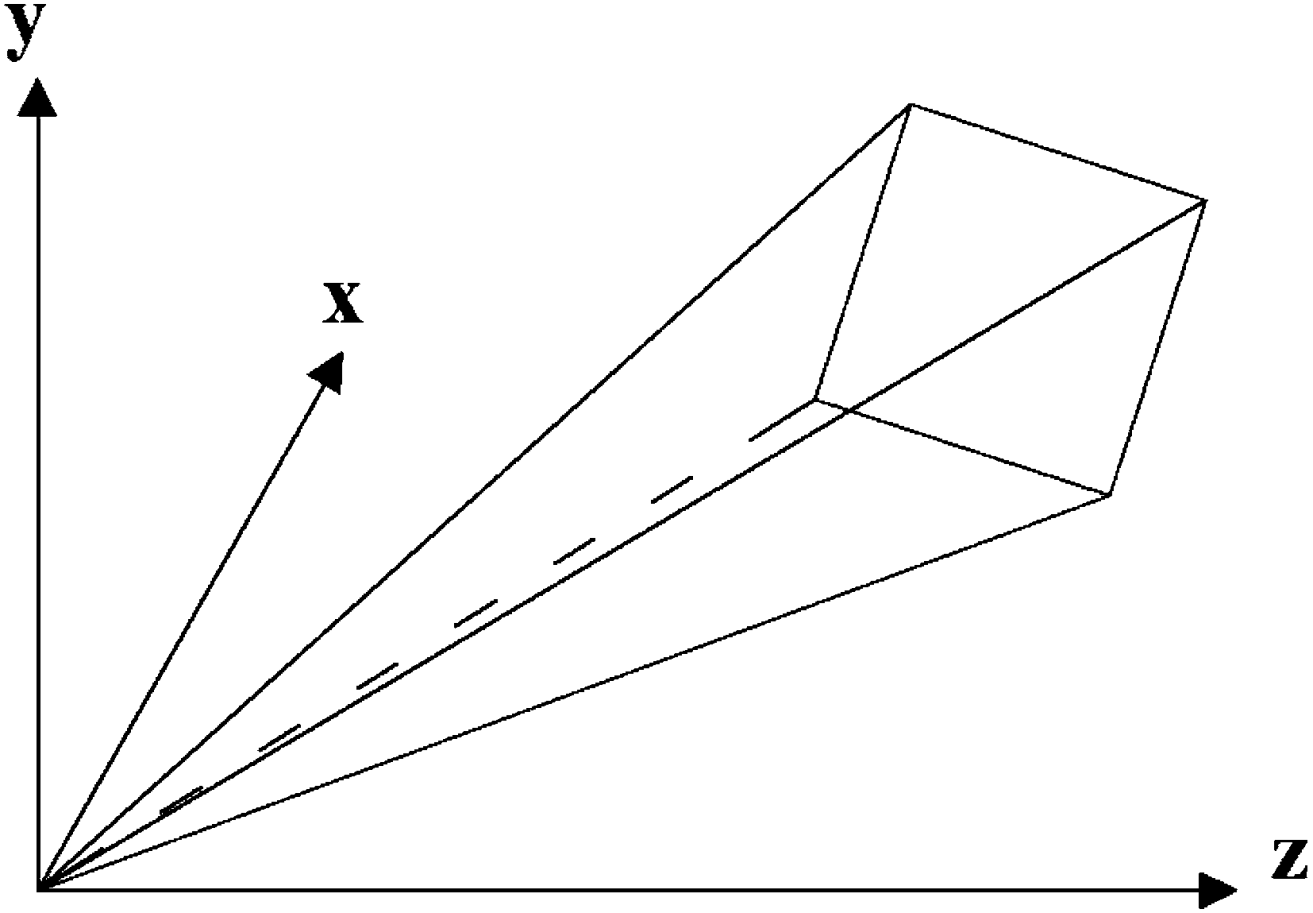
The target plane in the camera coordinate system.

The Bouguet method mentioned in reference^46^ is used to extract the image coordinates of four corner points in the black square pattern, as shown in Fig. 5. The plane equation of the target in the camera coordinate system can be solved by using the camera parameters, as shown in Fig. 6.

**Figure 7:**
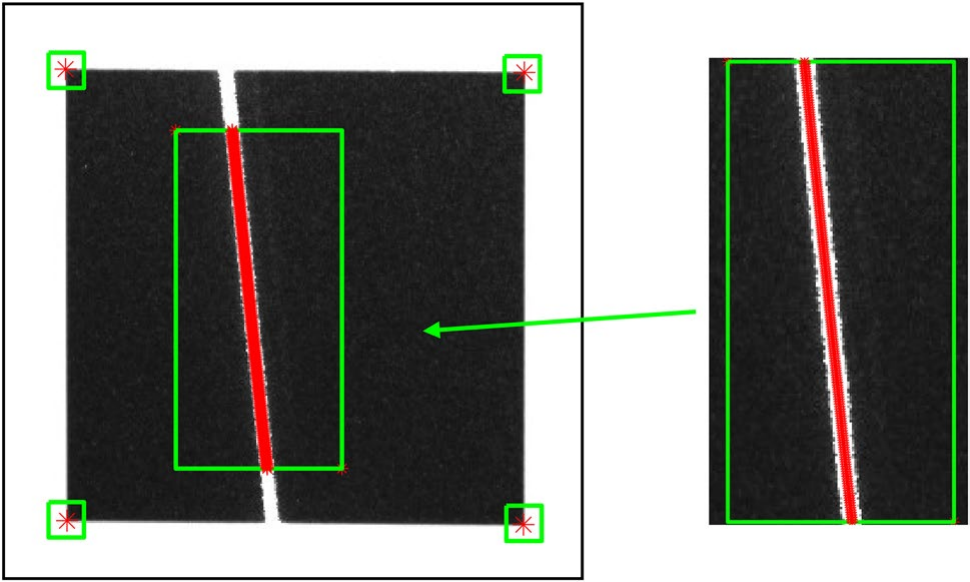
Extraction of the light strip center.

The sub-pixel coordinates of the center point of the light stripe can be accurately extracted using the Steger algorithm, as presented in the reference^47^. This algorithm, depicted in Fig. 7, enables precise localization of the center point with sub-pixel accuracy.

**Figure 8:**
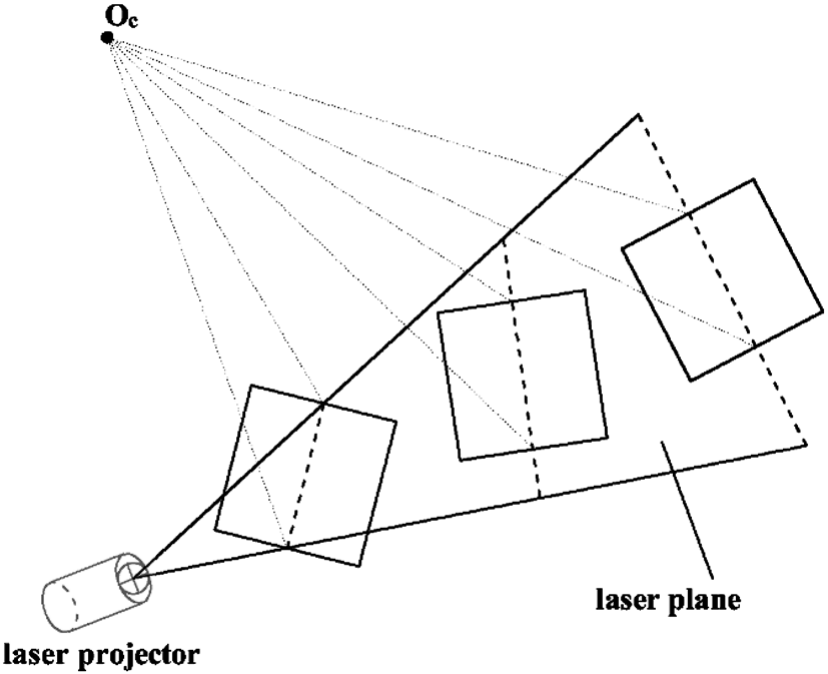
The fitting process of the laser plane.

The sub-pixel coordinates of the center points of light stripes in multiple images are projected into the camera coordinate system, and their 3D coordinates are calculated. Subsequently, a laser plane equation is fitted by these calculated 3D points. The fitting process is shown in Fig. 8.

The equation describing the laser plane can be expressed as follows:

1 $$Ax+By+Cz+1=0$$

In Eq. (1), $A$, $B$, and $C$ represent the coefficients of the laser plane equation. The values of these coefficients are determined through the calibration process discussed earlier. By fitting the 3D coordinates of the center points of the light stripes in multiple images and utilizing the camera and laser plane calibration information, the equations for both the initial scanning laser plane π and the positioning laser plane ∏ can be derived. The completion of this process signifies the successful initial calibration of the measurement system.

### The process of calculating three-dimensional coordinates

Camera calibration can establish the relationship between the pixel coordinates and the corresponding 3D world coordinates. The relationship is as follows:

2 $$\left[\begin{array}{c}{x}_{p}\\ {y}_{p}\\ 1\end{array}\right]=\left[\begin{array}{ccc}\alpha & \gamma & {u}_{0}\\ 0& \beta & {v}_{0}\\ 0& 0& 1\end{array}\right]\left[\begin{array}{c}{x}_{d}\\ {y}_{d}\\ 1\end{array}\right]$$

$$\left[\begin{array}{ccc}\alpha & \gamma & {u}_{0}\\ 0& \beta & {v}_{0}\\ 0& 0& 1\end{array}\right]=A$$, which is the camera’s intrinsic matrix, where $\alpha$ and $\beta$ represent the scale factors along the u and v axes of the image pixel coordinate system, respectively. $\gamma$ represents the non-orthogonality factor between the two coordinate axes of the pixel plane. ${(u}_{0},{v}_{0})$ denotes the coordinates of the intersection point between the camera’s optical axis and the image plane in the pixel coordinate system.

3 $$\left[\begin{array}{c}{x}_{u}\\ {y}_{u}\end{array}\right]=\left(1+{k}_{1}{r}^{2}+{k}_{2}{r}^{4}\right)\left[\begin{array}{c}{x}_{d}\\ {y}_{d}\end{array}\right]+\left[\begin{array}{c}2{p}_{1}{x}_{d}{y}_{d}+{p}_{2}\left({r}^{2}+2{x}_{d}^{2}\right)\\ {p}_{1}\left({r}^{2}+2{y}_{d}^{2}\right)+2{p}_{2}{x}_{d}{y}_{d}\end{array}\right]$$

Here, $r=\sqrt{{x}_{d}^{2}+{y}_{d}^{2}}$, where $\left({x}_{d},{y}_{d}\right)$ represents the actual image coordinates of a point in the image physical coordinate system. $\left({x}_{p},{y}_{p}\right)$ represents the pixel coordinates of the same point in the image pixel coordinate system. $({k}_{1},{k}_{2},{p}_{1},{p}_{2})$ are the distortion coefficients of the camera lens. ${k}_{1}$ and ${k}_{2}$ represent the coefficients of the radial distortion function in the image physical coordinate system, while ${p}_{1}$ and ${p}_{2}$ represent the coefficients of the tangential distortion function in the image physical coordinate system.

4 $$\left[\begin{array}{c}{x}_{u}\\ {y}_{u}\end{array}\right]=\frac{1}{{Z}_{c}}\left[\begin{array}{c}{X}_{c}\\ {Y}_{c}\end{array}\right]$$

where $({x}_{u},{y}_{u})$ represents the ideal image coordinates of a point in the image physical coordinate system, and $({X}_{c},{Y}_{c},{Z}_{c})$ represents the coordinates of a point in the camera coordinate system.

For each extracted two-dimensional pixel coordinate $({x}_{p},{y}_{p})$ in the image, the mapping relationship between the calculated coordinates using Eqs. (2) and (3), and the distortion-corrected coordinates $({x}_{u},{y}_{u})$ can be expressed as follows:

5 $$({x}_{u},{y}_{u})=f({x}_{p},{y}_{p})$$

Based on Eq. (4), the three-dimensional coordinates $({X}_{c},{Y}_{c},{Z}_{c})$ can be expressed in terms of the coordinate ${Z}_{c}$ and the function f as follows:

6 $${X}_{c}={Z}_{c}\cdot {x}_{u}={Z}_{c}\cdot f({x}_{p})$$

7 $${Y}_{c}={Z}_{c}\cdot {y}_{u}={Z}_{c}\cdot f({y}_{p})$$

The three-dimensional coordinates $({X}_{c},{Y}_{c},{Z}_{c})$ can be calculated using the known equation of the light plane. Substituting the three-dimensional coordinates into Eq. (1) yields the following formula:

8 $$A{X}_{c}+B{Y}_{c}+C{Z}_{c}+1=0$$

Based on Eqs. (5), (6), (7), and (8), the formula for three-dimensional coordinates is derived as follows:

9 $$\left\{\begin{array}{c}{X}_{c}=-f({x}_{p})/(A\cdot f({x}_{p})+B\cdot f({y}_{p})+C)\\ {Y}_{c}=-f({y}_{p})/(A\cdot f({x}_{p})+B\cdot f({y}_{p})+C)\\ {Z}_{c}=-1/(A\cdot f({x}_{p})+B\cdot f({y}_{p})+C)\end{array}\right.$$

## Real-time calibration algorithm of scanning laser plane equation

During the scanning process, the spatial equation of the scanning laser plane undergoes continuous changes, necessitating real-time calibration. In this section, we propose a real-time calibration algorithm for the scanning laser plane equation. This algorithm consists of two steps: first, the sub-pixel positioning method for light stripe intersection points, and second, solving the spatial equations of the scanning laser plane.

### Sub-pixel positioning method of the light stripe intersection point

**Figure 9:**
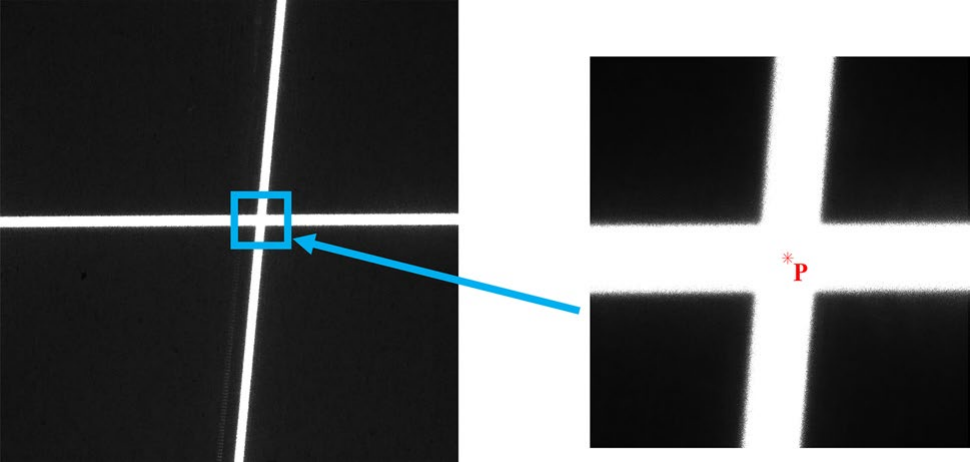
Laser intersection area and the center point P of the intersection area.

The accurate determination of light stripe intersection points is essential for precise calibration. In this paper, the center point of the intersection area between two light stripes is defined as the light stripe intersection point, as shown in Fig. 9.

**Figure 10:**
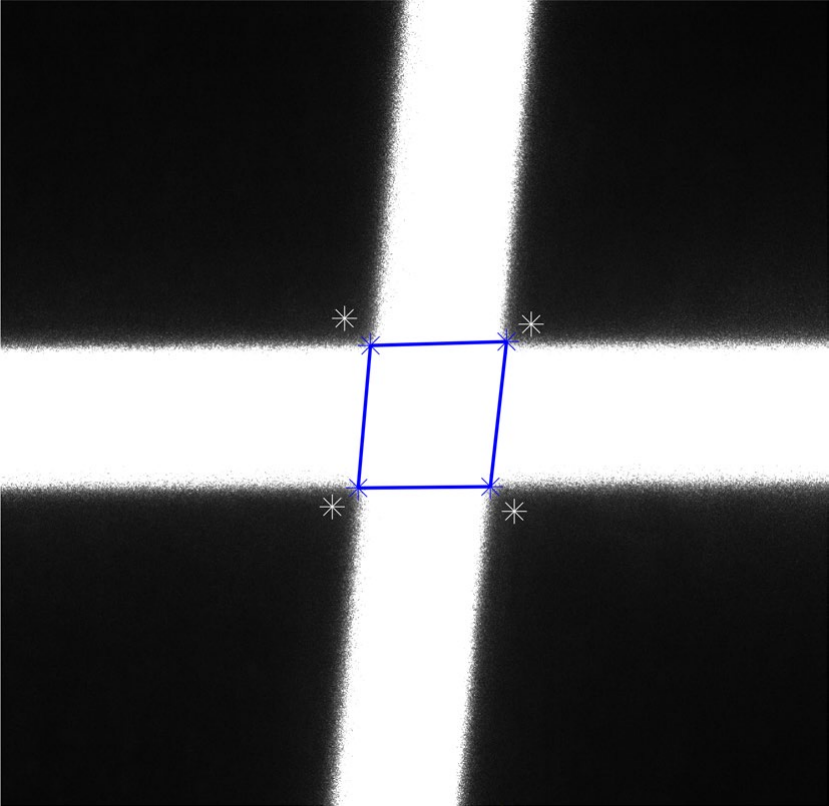
Quadrilateral formed by corner points.

As shown in Fig. 10, the intersecting area can be regarded as a closed quadrilateral formed by connecting the corner points so that the center point can be typically considered as the intersection point. However, due to the influence of lens distortion, the quadrilateral often becomes irregular, causing the geometric center to deviate from the true intersection point of its diagonals. To locate the coordinates of the intersection point, a sub-pixel positioning algorithm for the light stripe intersection points is given in this section.

#### Positioning of corner points

**Figure 11:**
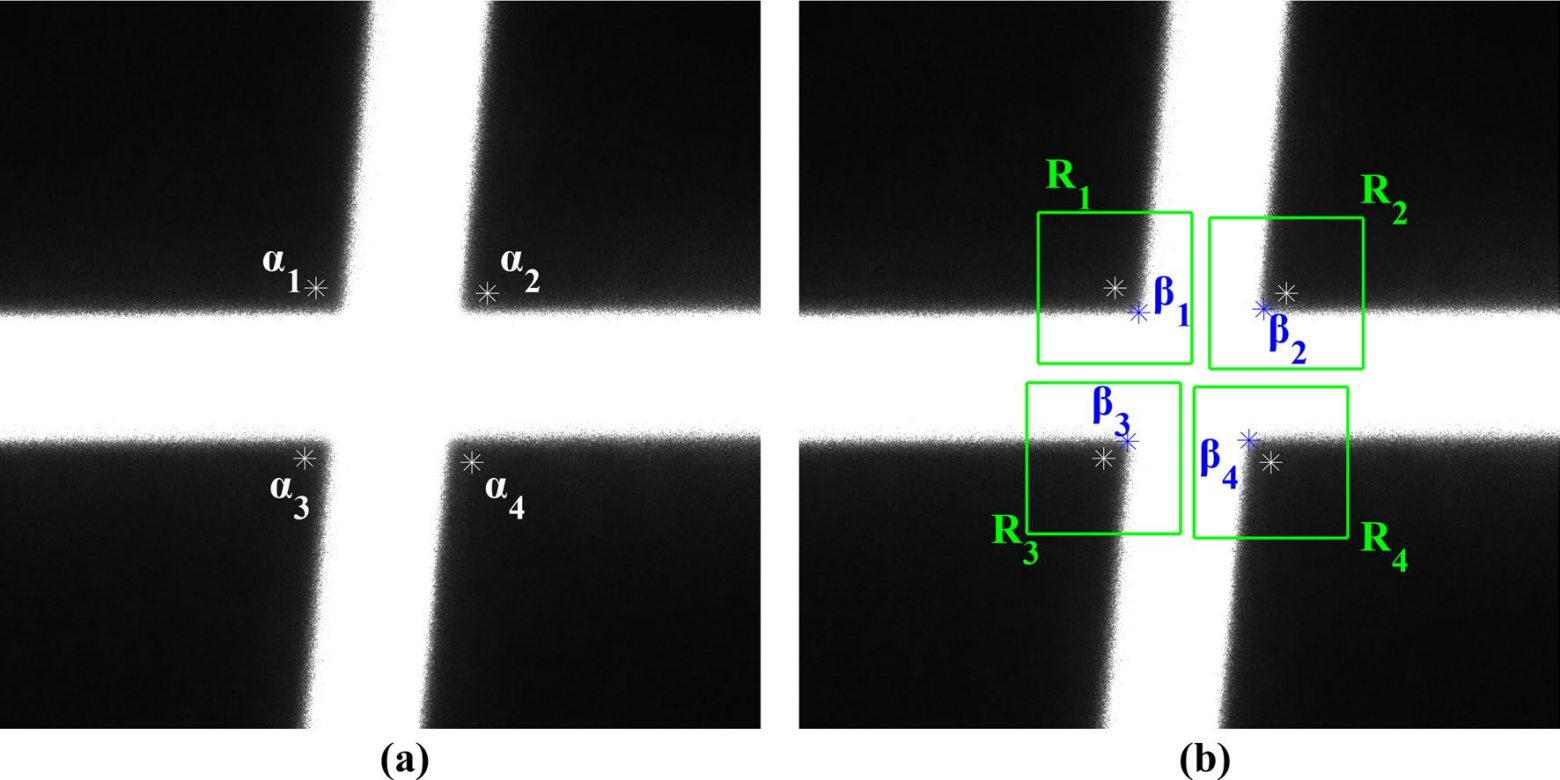
Detection of corner points, (**a**) pixel-level corner points ${\alpha }_{1}$, ${\alpha }_{2}$, ${\alpha }_{3}$, and ${\alpha }_{4}$, (**b**) sub-pixel detection area R_i_, and subpixel-level corner points ${\beta }_{1}$, ${\beta }_{2}$, ${\beta }_{3}$, and ${\beta }_{4}$.

Using the Harris corner point detection algorithm, the pixel coordinates of the corner points ${\alpha }_{1}$, ${\alpha }_{2}$, ${\alpha }_{3}$, and ${\alpha }_{4}$ are determined within the laser intersection area, as shown in Fig. 11a. The initial positioning of pixel-level corner points in the area is achieved. To further improve the accuracy of coordinates, a sub-pixel detection area R_i_ is defined around the pixel coordinates of each corner point. Sub-pixel corner point detection is further performed in each detection area R_i_ to obtain the corresponding sub-pixel coordinates. The resulting sub-pixel corner points ${\beta }_{1}$, ${\beta }_{2}$, ${\beta }_{3}$, and ${\beta }_{4}$ are shown in Fig. 11b.

#### Positioning of the center point

**Figure 12:**
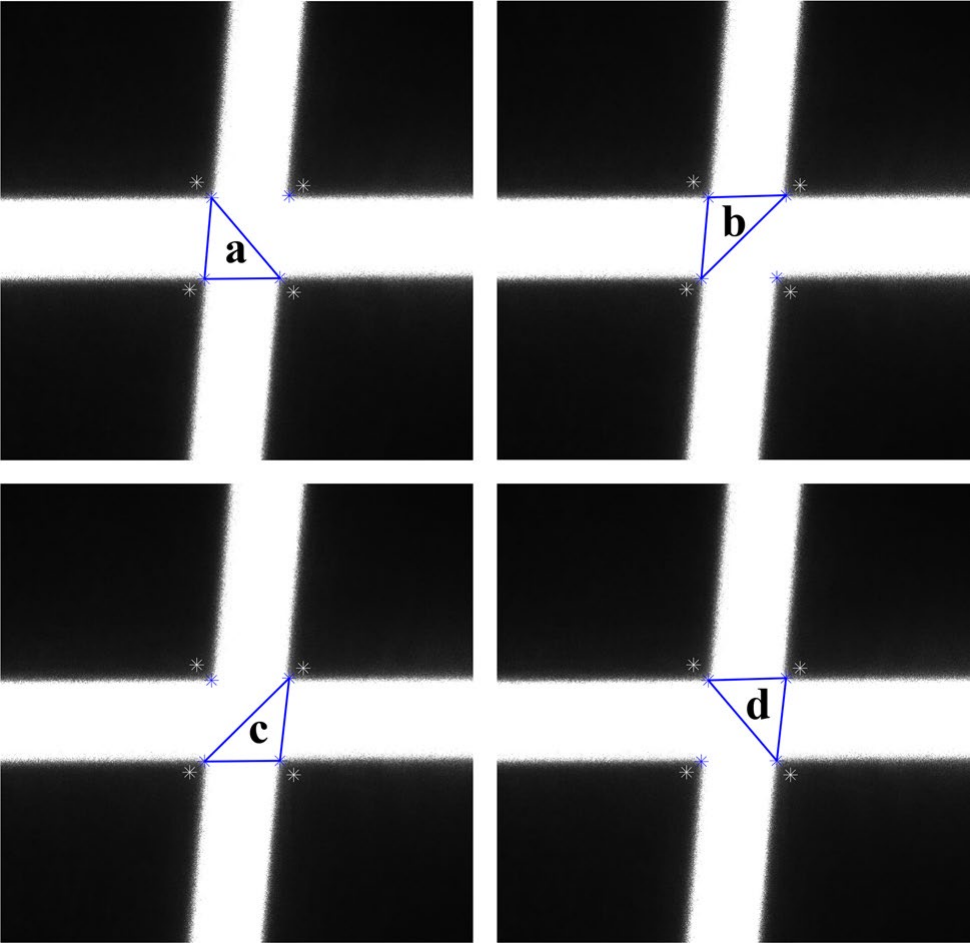
4 triangles a, b, c, and d.

An irregular quadrilateral can be divided into two triangles by one of its diagonals. It is known that the line connecting the barycenters of these two triangles passes through the geometric center of the quadrilateral. Exploiting this geometric property, we can obtain four triangles by connecting the two diagonal lines of the irregular quadrilateral determined by the connecting lines of the four points at the new position shown in Fig. 11b, as shown in Fig. 12. Consequently, the task of determining the geometric center of an irregular quadrilateral is transformed into solving the barycenters based on the properties of triangles.

**Figure 13:**
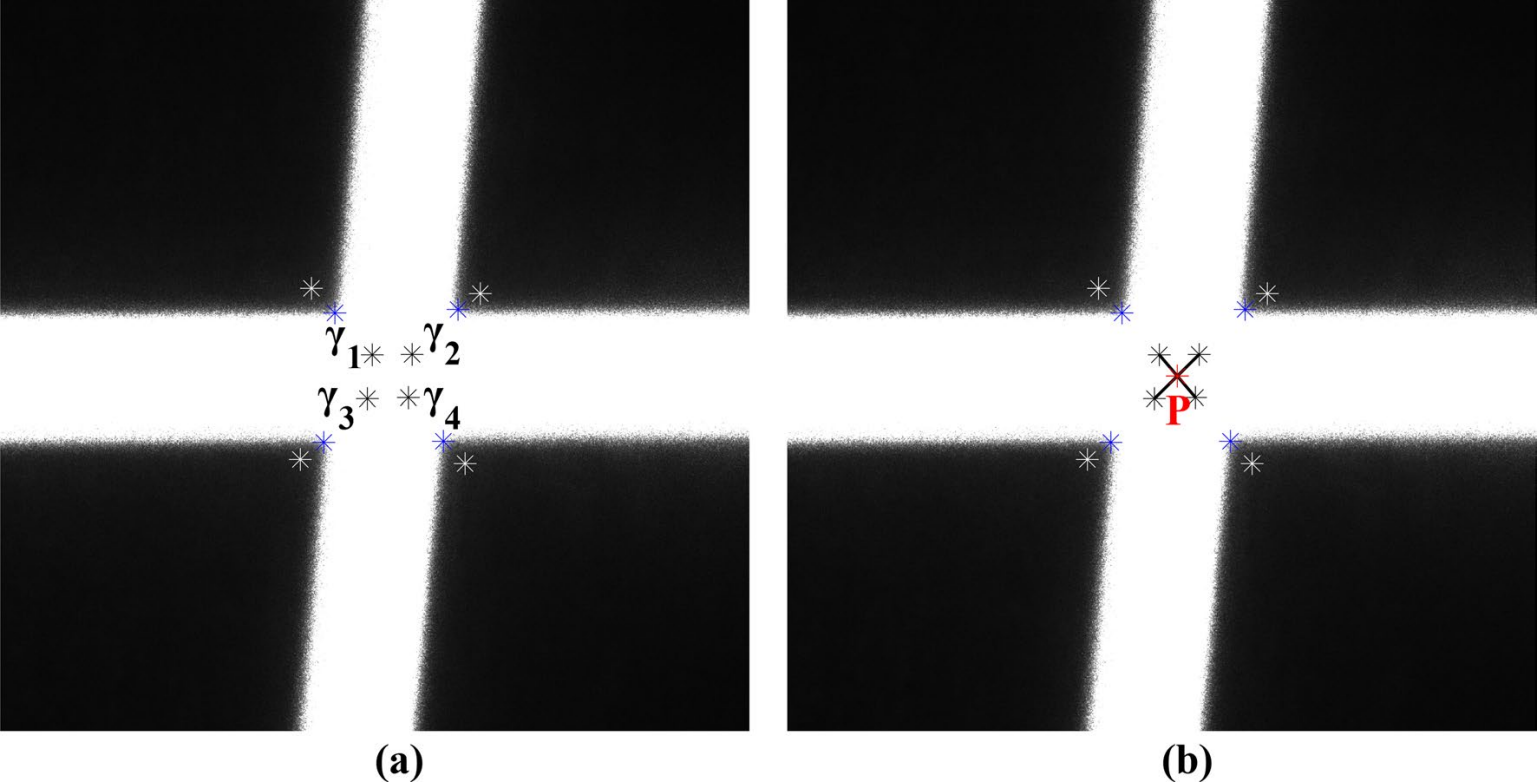
Detection of the center point, (**a**) barycentric points ${\gamma }_{1}$, ${\gamma }_{2}$, ${\gamma }_{3}$, and ${\gamma }_{4}$ of four triangles, (**b**) the center point P of the light stripe intersection area.

The barycentric coordinates of each triangle can be calculated using the sub-pixel coordinates of the corner points. This allows us to determine the positions of the barycentric points, denoted as ${\gamma }_{1}$, ${\gamma }_{2}$, ${\gamma }_{3}$, and ${\gamma }_{4}$, for the four triangles, as shown in Fig. 13a.

The barycentric points ${\gamma }_{1}$ and ${\gamma }_{4}$ of the triangles can be connected to form one line segment, while ${\gamma }_{2}$ and ${\gamma }_{3}$ can be connected to form another line segment. The intersection point of the two line segments represents the geometric center of the irregular quadrilateral and corresponds to the center point P of the light stripe intersection area, as shown in Fig. 13b.

### Solution of scanning laser plane spatial equation

In the measurement process, the scanning light stripe intersects with the positioning light stripe continuously, resulting in the intersection point lying in both planes. The sub-pixel coordinates of the light stripe intersection point can be projected into the camera coordinate system to obtain its spatial coordinates $(X,Y,Z)$ by using the calibration information of the positioning laser plane.

When the scanning laser plane moves along a fixed orientation, it remains parallel to the initial scanning laser plane. Consequently, all scanning laser planes share the same plane normal vector. Assuming that the vector $\overrightarrow{n}=(A,B,C)$ is the initial normal vector of the scanning laser plane. The formula for calculating the new scanning laser plane equation is as follows:

10 $$A\left(x-X\right)+B\left(y-Y\right)+\mathrm{C}\left(z-Z\right)=0$$

The real-time spatial plane equation of the scanning laser plane can be solved by Eq. (10), and real-time calibration in the measurement process can be achieved.

## Experiment and results

**Figure 14:**
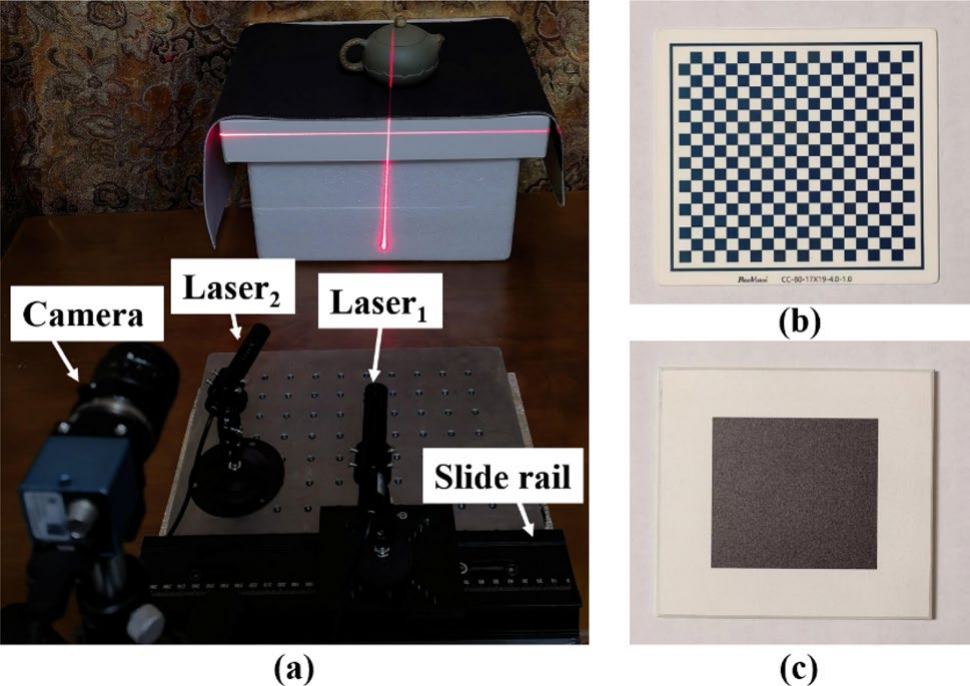
Related experimental equipment and environment, (**a**) camera, lasers, and slide rail, (**b**) checkerboard calibration board, (**c**) planar target.

The experimental part of this paper includes calibration experiment, scanning measurement and 3D reconstruction, and accuracy test. The experimental equipment used in the experiment includes a camera, two single-line lasers, a checkerboard calibration board, and a planar target with a black square pattern. Detailed models and parameters of the equipment can be found in Table 1, while Fig. 14 showcases the experimental equipment and environment.

**Table 1:** Detailed models and parameters of experimental equipment.

Experimental equipment	Model	Related parameters
Camera	MER-125-30UM@@ DaHeng, Beijing, China	Resolution: 1292 × 964 Lens focal length: 25 mm
Laser1 and Laser2	STR-660–20-CW-FL-L01-45-E-TX Coherent StingRay, CA, USA	Wavelength: 660 nm Power: 20 mW Fan Angle: 45° BeamDiameter: 1 mm
Checkerboard calibration board	CC-80–17 × 19–4.0–1.0 PointVision, Shenzhen, China	Number of squares: 17 × 19 Square size: 4 × 4 mm Accuracy: 1 μm
Planar target	ZXY-C 100-60 mm ZhiXing, Ningbo, China	Side length: 60 mm Accuracy: 1 μm

### Calibration experiment

**Figure 15:**
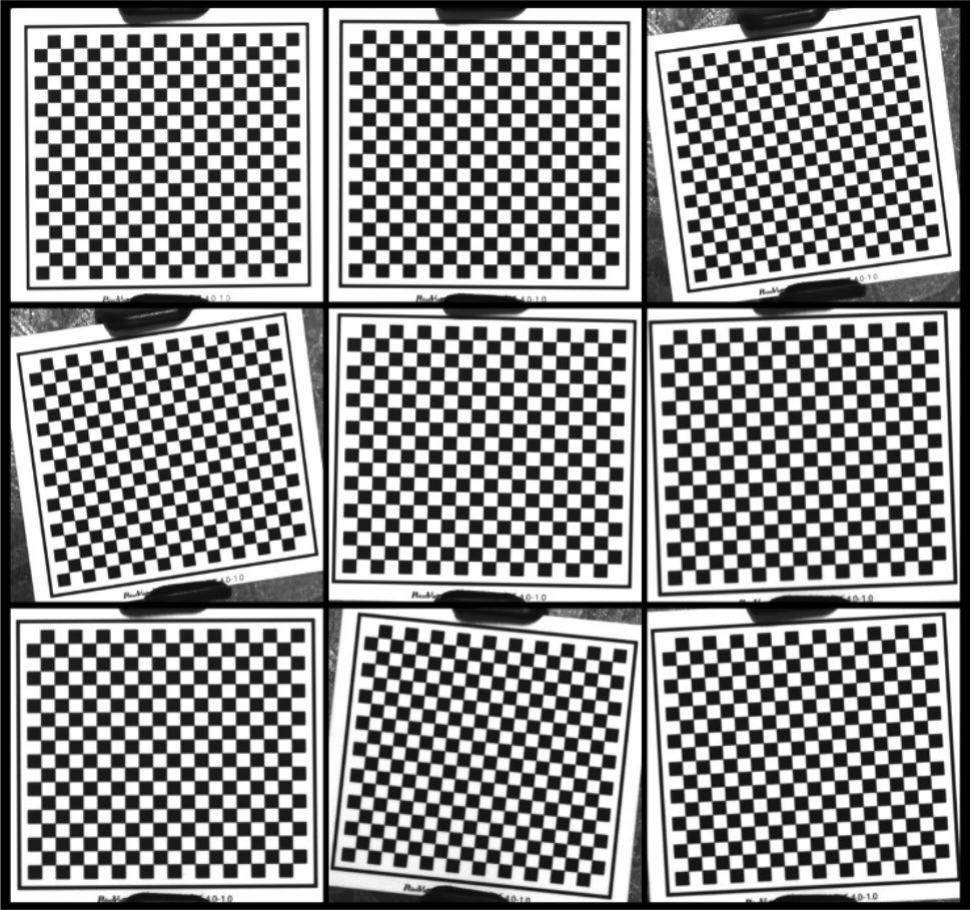
Images of the camera calibration.

In the calibration experiment, a total of nine images of the checkerboard calibration board were captured using the camera, as shown in Fig. 15. These images were then used to determine the camera’s internal parameter matrix and distortion coefficient, following the camera calibration model described in Camera calibration section. The resulting internal parameter matrix is represented as:

11 $$A=\left[\begin{array}{ccc}6719.91& -0.2285& 627.64\\ 0& 6720.29& 414.73\\ 0& 0& 1\end{array}\right]$$

Similarly, the distortion coefficient is represented as:

12 $$K=\left[\begin{array}{c}{k}_{1}\\ \begin{array}{c}{k}_{2}\\ {p}_{1}\\ {p}_{2}\end{array}\end{array}\right]=\left[\begin{array}{c}0.0259\\ \begin{array}{c}3.4770\\ 0.0010\\ 0.0003\end{array}\end{array}\right]$$

These parameters provide the necessary information to correct the camera’s lens distortion and accurately project the image coordinates onto the 3D world coordinates.

Then nine images of black square patterns with light strips at different positions were obtained using the camera. By applying the laser plane calibration method described in Laser plane section, the laser plane equations can be calculated. In this paper, the calibration is performed for two laser planes: the positioning laser plane and the initial scanning laser plane.

**Figure 16:**
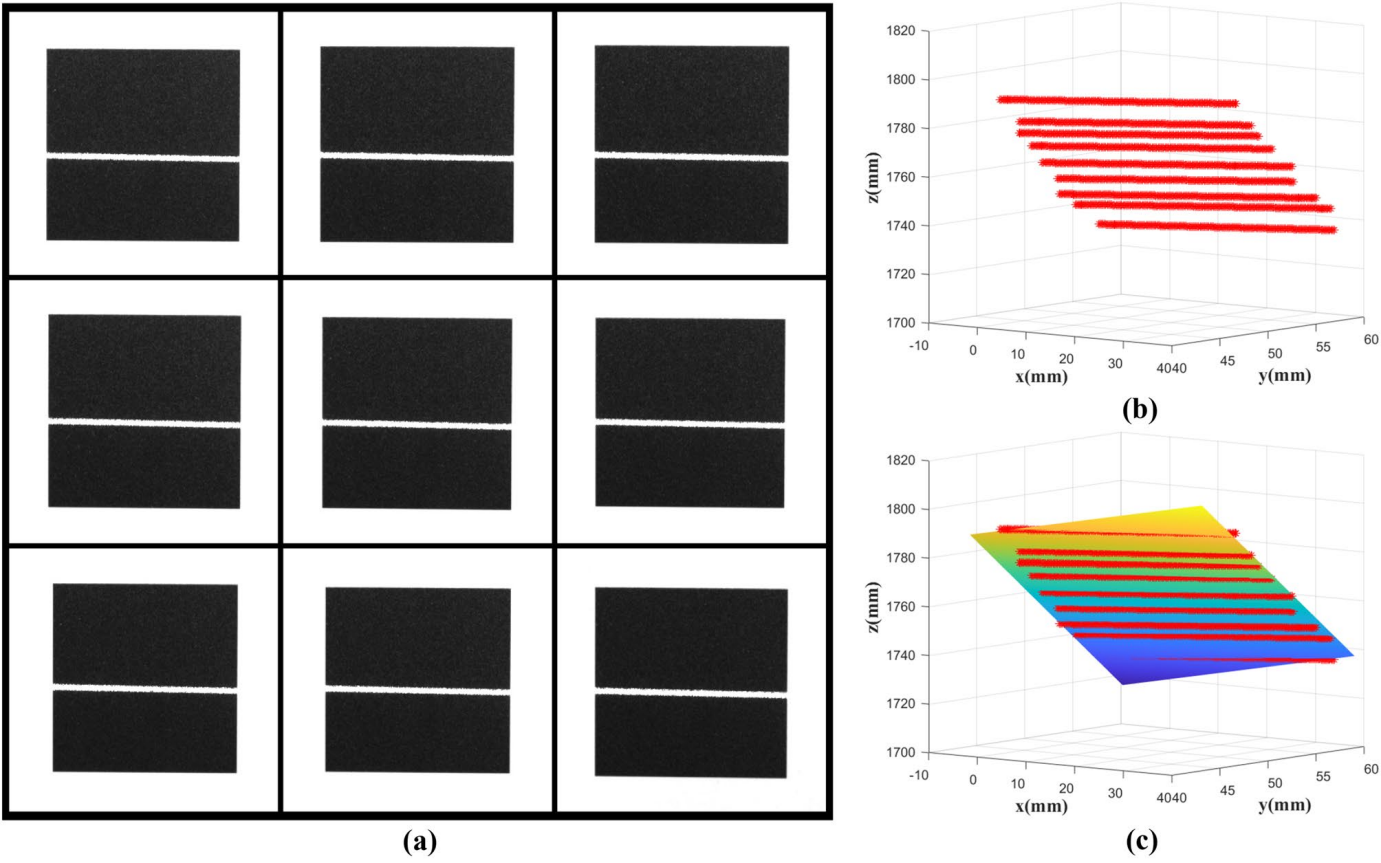
Positioning laser plane fitting, (**a**) calibration images, (**b**) 3D points of light stripe center, (**c**) fitting plane.

Figure 16 is a fitting process of the positioning laser plane. Figure 16a is nine images taken at different positions, and the 3D points in the center of the light stripe and the results of fitting the laser plane are shown in Fig. 16b and c. The calculated plane equation is:

13 $$0.2216x-2.2600y-0.4825z+1000=0$$

**Figure 17:**
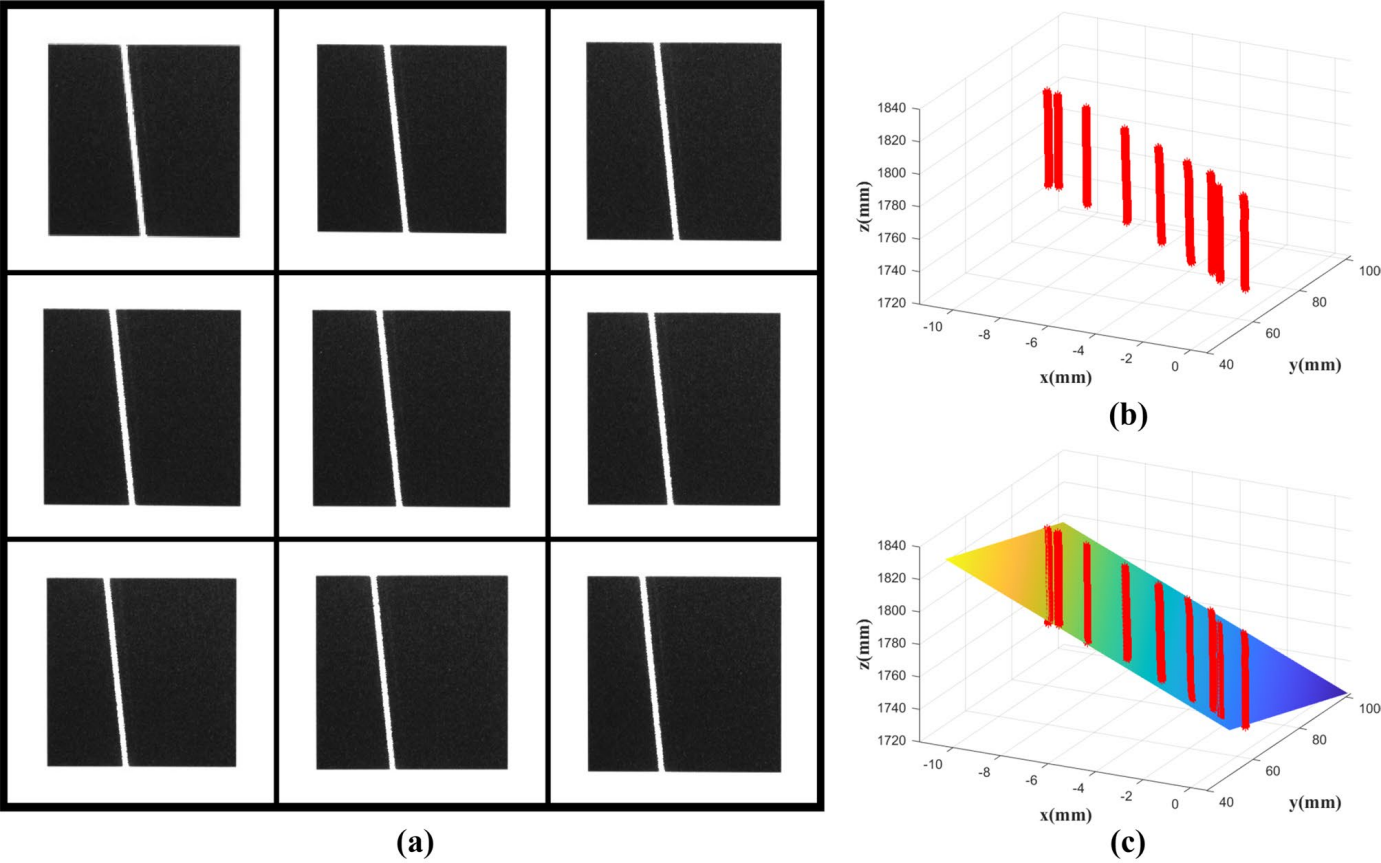
Initial scanning laser plane fitting, (**a**) calibration images, (**b**) 3D points of light stripe center, (**c**) fitting plane.

As shown in Fig. 17, the plane equation of the initial scanning laser plane is calculated by the same method, and the calculated plane equation is:

14 $$3.5450x+0.2901y+0.5630z-1000=0$$

### Scanning measurement and 3D reconstruction

In the scanning experiment, the 3D point cloud data of the measured object is obtained by scanning the object with the controlled scanning laser plane. The camera captures continuous images of the scanning light stripe’s motion, with a shooting rate of 50 frames per second. The scanning process lasts for 4.92 s, resulting in a total of 246 images, out of which 217 images are considered efficient.

**Figure 18:**
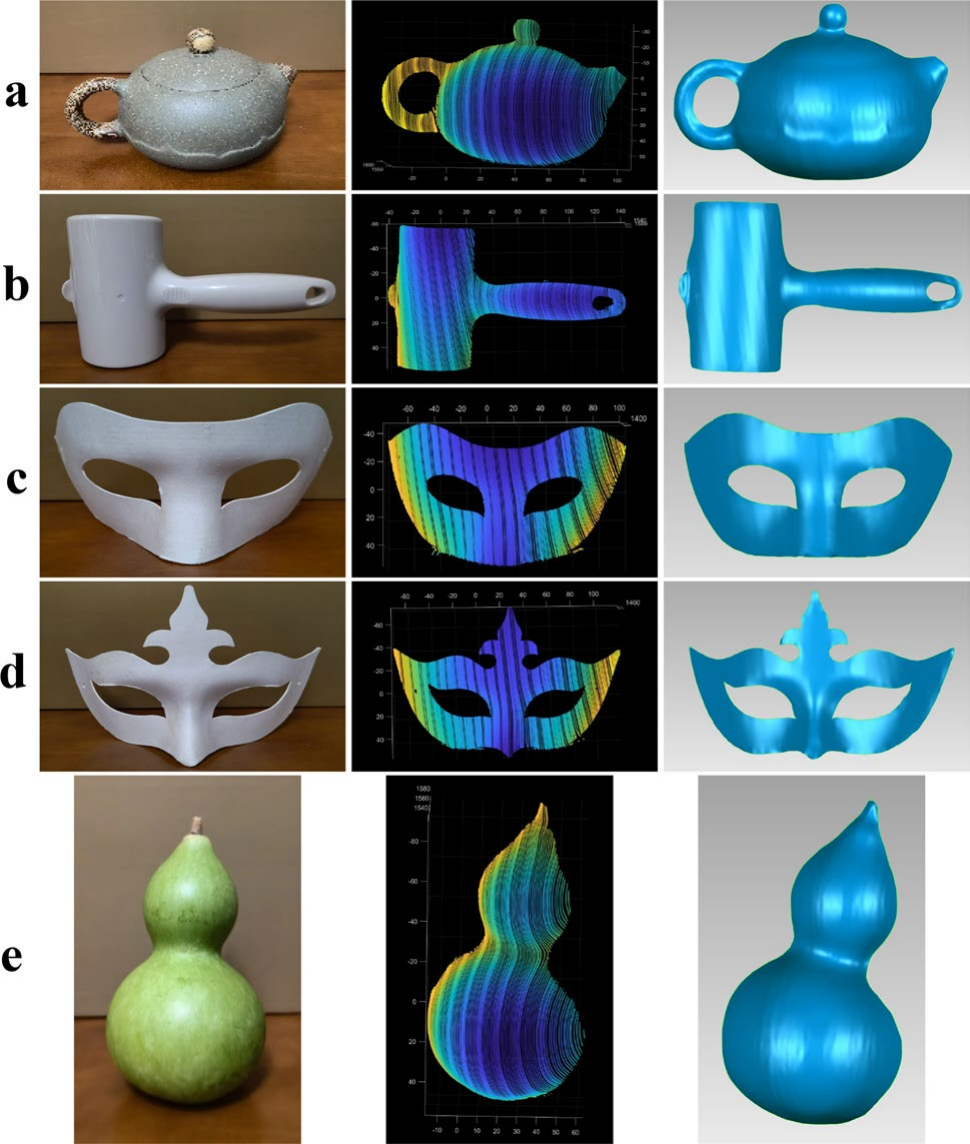
Scanning measurement and 3D reconstruction image set, a total of five sets of data (items a, b, c, d, and e, respectively). The sequence of each group of data is: original object, point cloud data, and 3D reconstruction surface shape.

The coordinates of the center point of each image are extracted by the Steger algorithm, and the 3D coordinates in the camera coordinate system are solved by combining the scanning laser plane equation of each frame to obtain the point cloud data of the image. Furthermore, the reverse engineering software Geomagic Wrap 2017 is utilized to fit the 3D surface of the point cloud data, resulting in the generation of a 3D reconstructed image. To evaluate the effectiveness of the system, five objects with different shapes are measured in this paper, as shown in Fig. 18.

### Accuracy test

**Figure 19:**
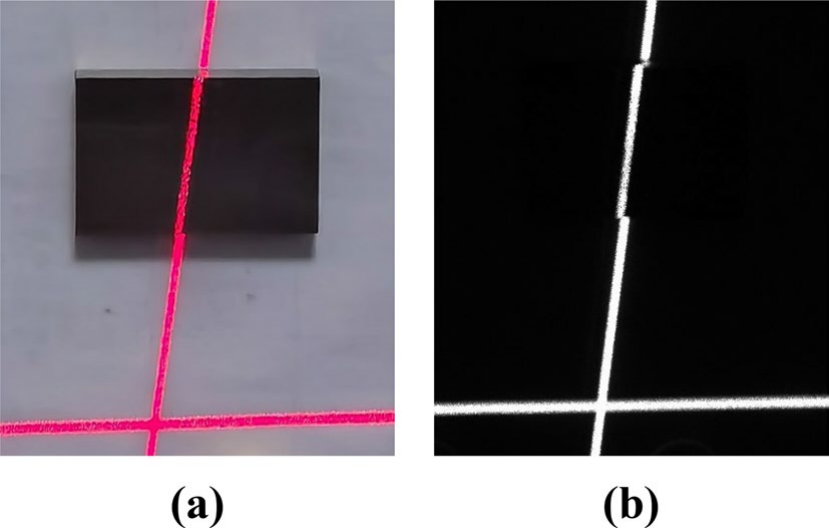
Gauge block measuring, (**a**) photographs, (**b**) images.

**Figure 20:**
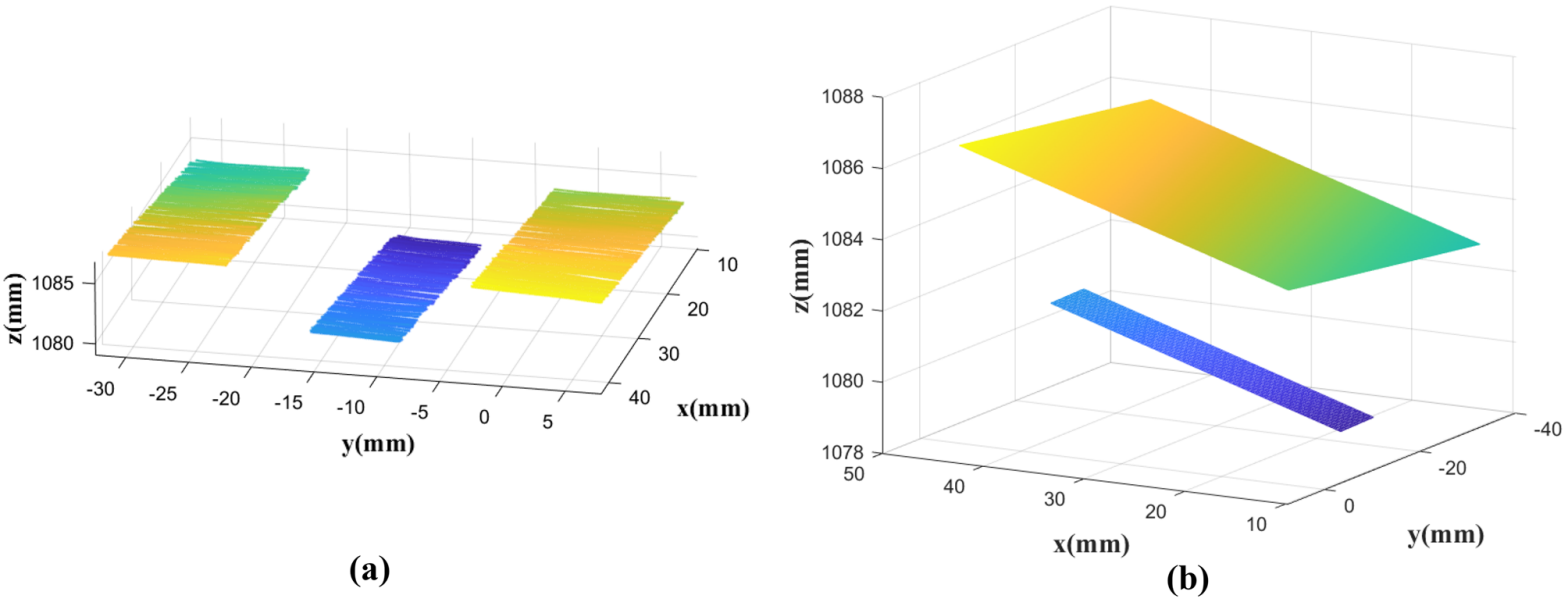
Gauge block measurements, (**a**) point cloud data for gauge block and background, (**b**) two parallel planes fitted from point cloud.

To test the accuracy of the structured light vision measurement system, rectangular gauge blocks with known thicknesses are measured in this paper. The precision of the gauge block is 1 μm. During the measurement process, a scanning light stripe is projected onto the surface of the gauge block. As shown in Fig. 19, the light stripes on the surface of the gauge block and the background plane are separated by the gauge block itself. Point cloud data of gauge block surface and background plane are extracted respectively, and two parallel planes can be obtained using the scanning measurement method, as shown in Fig. 20. The distance between these two parallel planes is the measured value of the thickness of the gauge block. To assess the accuracy of the measurement system, the actual thickness of the gauge block is considered as the reference value. The difference between the measured thickness obtained from the structured light vision measurement system and the actual thickness of the gauge block is analyzed to determine the error of the measurement system.

To ensure the reliability of the experimental data, this study utilized ten gauge blocks of varying specifications. The known thicknesses of these gauge blocks were as follows: 1 mm, 3 mm, 5 mm, 10 mm, 15 mm, 20 mm, 25 mm, 30 mm, 40 mm, and 50 mm. Each gauge block was subjected to 20 individual measurements, from which the mean measurement value was determined. Subsequent to this, the Mean Absolute Error (MAE), Average Error (AE), and Root Mean Square Error (RMSE) were computed to evaluate the precision of the measurement system.

The MAE serves as an indicator of the average magnitude of systematic errors inherent to the system. AE, on the other hand, considers the directionality of errors, while the RMSE provides a nuanced reflection of the measurement errors. The measurement outcomes and their respective error values are delineated in Table 2. Upon analysis of the data presented in Table 2, it becomes evident that for the gauge block with a maximal thickness of 50 mm, the MAE is approximately 0.0793 mm, the AE is around -0.0016 mm, and the RMSE is roughly 0.0974 mm. These results affirm the exemplary accuracy of the measurement system devised in this investigation.

**Table 2:** The measurement results presented in this paper (mm).

Gauge block number	Known thickness	Mean measurement value	MAE	AE	RMSE
1	1.0000	0.9909	0.0414	− 0.0091	0.0505
2	3.0000	2.9913	0.0341	− 0.0087	0.0421
3	5.0000	5.0024	0.0319	0.0024	0.0395
4	10.0000	10.0041	0.0368	0.0041	0.0477
5	15.0000	14.9943	0.0367	− 0.0057	0.0449
6	20.0000	20.0173	0.0479	0.0173	0.0589
7	25.0000	24.9839	0.0545	− 0.0161	0.0682
8	30.0000	30.0374	0.0616	0.0374	0.0734
9	40.0000	40.0002	0.0654	0.0002	0.0845
10	50.0000	49.9984	0.0793	− 0.0016	0.0974

A comparative evaluation of measurement accuracy was conducted by employing methodologies analogous to those found in other references. Specifically, in reference^22^, gauge blocks with standardized thicknesses akin to those in our study, specifically 14.873 mm and 29.746 mm, exhibited AE values of − 0.039 mm and 0.139 mm, respectively. In contrast, our study yielded AE values of − 0.0057 mm and 0.0374 mm for gauge blocks with thicknesses of 15 mm and 30 mm. Furthermore, in reference^23^, the AE values for measurements of 1 mm and 3 mm gauge blocks stood at 0.0222 mm and 0.0116 mm, respectively. Contrarily, our study documented AE values of -0.0091 mm and − 0.0087 mm for the corresponding gauge blocks. Based on this empirical data, the methodology employed in our study demonstrates reduced measurement errors, highlighting its superiority over the methods proposed in references^22,23^.

**Figure 21:**
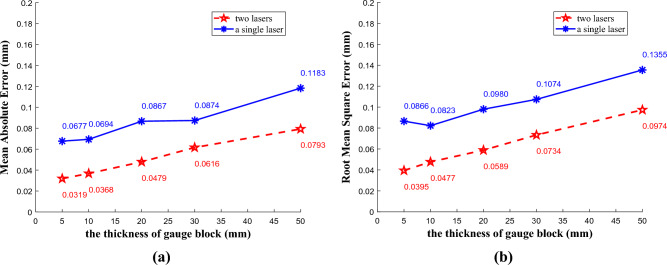
Comparison between two lasers method and a single laser method, (**a**) mean absolute error, (**b**) root mean square error.

In addition, this article also measured the accuracy of a single laser. The experimental setup has been adjusted to ensure that the single-line structured light is perpendicular to the surface of the test object, and the laser direction corresponds to the scanning direction of the laser plane along the sliding rail. Each gauge block was also measured 20 times. The measurement results using a single laser are shown in Table 3. At the same time, in comparison with the methods presented in this article, we selected 5 representative gauge blocks and displayed the differences by comparing MAE and RMSE. The results are shown in Fig. 21. The experimental results show that compared to using a single laser, the method of using two lasers in this paper has lower errors and improves measurement accuracy and stability to a certain extent.

**Table 3:** The measurement results using a single laser (mm).

Gauge block number	Known thickness	Mean measurement value	MAE	AE	RMSE
1	1.0000	0.9990	0.0796	− 0.0010	0.0939
2	3.0000	2.9844	0.0676	− 0.0156	0.0859
3	5.0000	5.0121	0.0677	0.0121	0.0866
4	10.0000	10.0169	0.0694	0.0169	0.0823
5	15.0000	15.0310	0.0663	0.0310	0.0827
6	20.0000	20.0146	0.0867	0.0146	0.0980
7	25.0000	24.9805	0.1119	− 0.0195	0.1410
8	30.0000	30.0148	0.0874	0.0148	0.1074
9	40.0000	40.0455	0.1081	0.0455	0.1256
10	50.0000	49.9958	0.1183	− 0.0042	0.1355

## Discussion

In this paper, we presented a novel approach for 3D surface shape measurement using a double-line combined structured light 3D vision measurement system. The system integration of cameras, two single-line lasers, and a slide rail enables rapid and accurate measurements, eliminating the need for pre-determining scanning directions and step sizes. The real-time calibration during the scanning process, achieved through computing the scanning laser plane equation using 3D coordinates of light stripe intersection points and the initial light plane position, is an innovative aspect of our work.

Our experimental results demonstrate the effectiveness of the proposed system in accurately reconstructing 3D surfaces while maintaining stability and data accuracy. The validation experiments included calibration, scanning measurements, and accuracy test. When comparing our method with existing techniques, our approach exhibited excellent accuracy. This highlights the potential impact of our methodology in addressing the limitations of other methods and enhancing measurement precision. Moreover, in contrast to traditional single-line structured light measurement methods, our system exhibited reduced errors and improved measurement accuracy and stability.

Given our current apparatus, we suggest the following measurement parameters: System working distance: 0.5 m-2.4 m. The camera’s scanning angular range (H × W) is 8.2° × 10.9°. The near-distance scanning range (H × W) is 71.7 mm × 95.4 mm, while the far-distance scanning range (H × W) is 344.2 mm × 457.9 mm. The capture volume size is 0.3748m^3^. By measuring gauge blocks of different sizes and after comprehensive analysis, the measurement error of this system is less than 1%.

In conclusion, our study introduces a novel solution to the field of structured light vision measurement. The double-line combined structured light 3D vision measurement system offers significant advantages over existing single-line methods, demonstrating its potential to overcome limitations and deliver accurate results. Future research could explore further applications and refinements to enhance the system’s performance and versatility.

## Conclusion

In this paper, a novel approach for 3D surface shape measurement using a double-line combined structured light 3D vision measurement system is proposed. The system utilizes essential equipment such as lasers, cameras, and a slide rail to enable fast and accurate measurement without the necessity of predetermining the scanning direction and motion step. During the scanning process, the real-time calculation of the scanning laser plane equation is solved by using the 3D coordinates of the light strip intersection point and the initial position of the light plane. Experimental results demonstrate the system’s ability to accurately reconstruct 3D surfaces while maintaining stability and data accuracy. In a word, the method proposed in this paper provides a new solution for structured light vision measurement and has a good effect in practical application.

## Data Availability

All data generated or analyzed during this study are included in this manuscript.
